# Academic Electronic Health Records in Undergraduate Nursing Education: Mixed Methods Pilot Study

**DOI:** 10.2196/26944

**Published:** 2021-04-27

**Authors:** Manal Kleib, Deirdre Jackman, Uirá Duarte Wisnesky, Shamsa Ali

**Affiliations:** 1 Faculty of Nursing University of Alberta Edmonton, AB Canada

**Keywords:** academic electronic health record, Lippincott DocuCare, simulation, nursing informatics education

## Abstract

**Background:**

Teaching students about electronic health records presents challenges for most nursing programs, primarily because of the limited training opportunities within clinical practice settings. A simulated electronic health record is an experiential, learner-centered strategy that enables students to acquire and apply the informatics knowledge needed for working with electronic records in a safe learning environment before the students have encounters with real patients.

**Objective:**

The aim of this study is to provide a preliminary evaluation of the Lippincott DocuCare simulated electronic health record and determine the feasibility issues associated with its implementation.

**Methods:**

We used one-group pretest-posttest, surveys, and focus group interviews with students and instructors to pilot the DocuCare simulated electronic health record within an undergraduate nursing program in Western Canada. Volunteering students worked through 4 case scenarios during a 1-month pilot. Self-reported informatics knowledge and attitudes toward the electronic health record, accuracy of computerized documentation, satisfaction, and students’ and educators’ experiences were examined. Demographic and general information regarding informatics learning was also collected.

**Results:**

Although 23 students participated in this study, only 13 completed surveys were included in the analysis. Almost two-thirds of the students indicated their overall understanding of nursing informatics as being fair or inadequate. The two-tailed paired samples *t* test used to evaluate the impact of DocuCare on students’ self-reported informatics knowledge and attitudes toward the electronic health record revealed a statistically significant difference in the mean score of knowledge before and after using DocuCare (before: mean 2.95, SD 0.58; after: mean 3.83, SD 0.39; *t*_12_=5.80, two-tailed; *P*<.001). There was no statistically significant difference in the mean scores of attitudes toward the electronic health record before and after using DocuCare (before: mean 3.75, SD 0.40; after: mean 3.70, SD 0.34; *t*_12_=0.39, two-tailed; *P*=.70). Students’ documentation scores varied from somewhat accurate to completely accurate; however, performance improved for the majority of students as they progressed from case scenarios 1 to 4. Both the faculty and students were highly satisfied with DocuCare and highly recommended its integration. Focus groups with 7 students and 3 educators revealed multiple themes. The participants shared suggestions regarding the DocuCare product customization and strategies for potential integration in undergraduate nursing programs.

**Conclusions:**

This study demonstrated the feasibility and suitability of the DocuCare program as a tool to enhance students’ learning about informatics and computerized documentation in electronic health records. Recommendations will be made to academic leadership in undergraduate programs on the basis of this study. Furthermore, a controlled evaluation study will be conducted in the future.

## Introduction

### Background

Electronic health records (EHRs) are an essential component of modern-day digitally connected health care. An EHR is a secure and integrated digital health technology that houses patients’ information and encounters with the health system. EHRs help improve access to health services, enhance the quality and safety of care, and increase the efficiency of the health system [1]. As the largest group of care providers, nurses are increasingly using digital health tools across practice settings [2,3]. Therefore, nursing schools must provide theoretical and technical knowledge related to these health information systems for nursing students [4,5]. This is particularly important given the growing concerns regarding the lack of preparedness in using health information systems among graduating students once they join the workplace [6-10]. However, teaching students about EHRs presents challenges for most schools of nursing, primarily because of the limited EHR training opportunities within practice settings and the complexity of educating a large number of students in busy and often complex clinical environments [5,11-13].

Simulated EHRs for academic purposes have been proposed as an innovative pedagogy to promote the acquisition of theoretical knowledge of informatics and the skills needed to use EHRs in a safe environment before encounters with real patients [14-25]. Incorporating these learning experiences within undergraduate nursing education expands the realm of effective teaching and learning practice, fulfills accreditation requirements, and ensures graduates have the required entry-to-practice competencies in nursing informatics upon exiting the program so that they are better prepared for practice in today’s digitally rich health care environment [3,26-28]. A simulated EHR is a learner-centered pedagogy grounded in a constructivist approach of experiential learning where learners are actively involved in the process of knowledge construction and reflective learning as opposed to passively receiving information [15,29,30]. Using case scenarios that mimic real-world clinical practice enables the students to learn how to use EHRs for care delivery, eg, identifying and interpreting patient data such as verifying medication accuracy, in a supervised environment before clinical encounters with real patients; thus, it improves learning outcomes and can contribute to promoting patient safety [20,30-36].

Simulated EHRs resemble electronic patient records used in practice settings [22,37]. A number of products are available for nurse educators to choose from including open-source EHR or electronic medical record (EMR) and vendor-operated EHR or EMR platforms [38]. Examples of open-source platforms include OpenEMR, WorldVistA, and OSCAREMR. Vendor-operated platforms are available through health information system technology vendors such as Cerner. These also are offered through publishing companies such as Lippincott DocuCare by Wolters Kluwer, EHR Tutor by Assessment Technologies Institute, SimChart by Elsevier, and NEEHR Perfect, now known as ehr^go^, by Archetype Innovations. Although these products have similar platforms, they vary in cost and functionality [38].

Research suggests that integrating simulated EHRs in prelicensure nursing education is beneficial to students’ learning and development of important educational outcomes [20,30,34]. Simulated EHRs help improve students’ critical thinking [18] and their ability to navigate EHRs and understand informatics concepts such as data management [19,39,40]. Other researchers found that simulated EHRs help enhance students’ skills in electronic documentation [24,41,42] and aid in the development of positive attitudes and perspectives about electronic records [43,44]. The integration of simulated EHRs also enhances students’ confidence and self-efficacy in using electronic records [23,44-46] and increases informatics knowledge and competency [16,40,47]. Despite these benefits, factors such as cost and faculty expertise continue to be major challenges in integrating simulated EHRs in nursing education [18,34,48,49].

At our university, curricular revisions presented an ideal opportunity to explore the integration of a simulated EHR within undergraduate nursing programs to expose students to health information technologies used in practice and to acquire the required entry-to-practice informatics competencies. This direction was enforced further by the introduction of Connect Care, a new EMR, in our health system. Although our students will have the opportunity to complete a user-training program before their clinical practicum in units that transitioned to Connect Care, these students have no previous exposure to hands-on practice with electronic records within their laboratory or simulation education, which is a gap in our curriculum. To support students’ learning and application of informatics knowledge that is currently taught in theory courses and the computerized documentation introduced in the new curriculum across all clinical courses, we reviewed a number of simulated EHR solutions considering the benefits and value in meeting the students’ and program’s needs [16,18,37,38]. We opted for the Lippincott DocuCare simulated EHR because it is user friendly and is aligned with the V-Sim resources currently used in our simulation laboratory. In addition, the cost of DocuCare for an individual student’s web access at US $100 for 12 months (negotiable when based on an institutional purchase plan) provided additional support for choosing this product over others. The next step of our evaluation was to obtain feedback from students and faculty on the suitability of this product for integration into our programs.

### Research Questions

This pilot study aimed to provide a preliminary evaluation of the Lippincott DocuCare program and to determine the feasibility issues associated with its implementation. The following research questions were examined:

1. Is there a significant difference in the mean scores of self-reported informatics knowledge and attitudes toward the EHR before and after using DocuCare?
2. What is the accuracy of students’ electronic documentation?
3. Are students satisfied with using DocuCare as a learning tool?
4. How do students and educators describe their experiences using DocuCare as a learning tool to improve computerized documentation and overall informatics competency?

## Methods

### Design and Sample

In this mixed method pilot study, a quasi-experimental one-group pretest-posttest design using short surveys was used to answer research questions 1 to 3, which were relevant to students’ learning and satisfaction with DocuCare. Focus group interviews with students and educators were used to answer research question 4 [50]. A convenience sample from third- and fourth-year nursing students enrolled in undergraduate nursing programs in the Faculty of Nursing was invited to participate in this study because, at this level of education, they would have had some exposure to clinical practice and would have accumulated sufficient theoretical knowledge. Nursing educators involved in laboratory and clinical teaching within the undergraduate programs were also invited to participate.

### Study Procedures

Students were recruited through an announcement supplemented with an information sheet on the e-class site, inviting students registered in a number of third- and fourth-year courses to express interest in participating by contacting the researchers. A list of interested participants was then compiled, and the volunteering students were enrolled in the study on a first-come first-serve basis.

Interested student participants were contacted via email and asked to complete a survey (pretest), as described later, and to indicate their availability for attending an in-person orientation session to have a practice demo, using a scavenger hunt exercise, on how to use DocuCare. Two dates were provided with the option for an evening and weekend meeting time to accommodate students’ schedules. At the same time, a total of 50 access codes were requested from DocuCare for use by students and educators in this study at no cost. Students who completed the pretest survey were then contacted and given the unique DocuCare access codes. As only a few students were able to attend the in-person orientation, a supplementary written guide with step-by-step directions and URL links to DocuCare publisher training videos on how to use the program was provided as a reference when they used DocuCare on their own.

The pretest-posttest survey included 18 items organized into 3 sections. This survey was administered to students at the beginning of the pilot (pretest) and used again as a posttest at the end of the pilot. Section 1 included 8 questions related to demographic and general information: program, year of study, opportunities for learning about informatics competencies in theory and laboratory training, exposure to electronic documentation in clinical sites, and support resources available to students when learning about computerized documentation. Section 2 included 5 Likert-type scale items (strongly disagree to strongly agree) that measured self-reported informatics knowledge using the entry-to-practice informatics competency indicators relevant to documentation and data management. These indicators have been validated in another study (Cronbach α=.93) [51]. Section 3 included 5 Likert-type scale items (strongly disagree to strongly agree) that measured nurses’ attitudes and dispositions toward the EHRs (Cronbach α=.77) [52]. A high score on this scale indicates a positive disposition, and a low score indicates a negative disposition. Research Electronic Data Capture (REDCap; Vanderbilt University) software was used to administer the data collection tools [53].

During the pilot, which was conducted over approximately 1 month, the participating students were asked to work on 4 patient case scenarios within the DocuCare platform, 1 scenario per week, or as their schedule permitted. Each case scenario required 1 to 2 hours on average, during which time the students needed to find information required for care planning, electronically document the care provided, and submit their completed work to the instructor for assessment. A standardized answer key for each scenario and a marking rubric, developed for the purpose of this study, were used to enhance objectivity and consistency in marking students’ submissions in DocuCare. For each case scenario, the students’ submissions were marked against the rubric using a rating scale from 1 to 4 (1=inaccurate, 2=somewhat accurate, 3=fairly accurate, and 4=completely accurate). A total score of 16 was tallied for the 4 case scenarios for each student. Students’ previous documentation skills were not tested at baseline. Owing to their workload, laboratory educators were not available to participate in marking students’ submissions. Instead, a master’s-level graduate student was hired to assist with the project. The graduate student marked the students’ submissions, provided feedback, tracked the students’ progress, and responded to questions they had during the pilot. These marks were not included in academic grades. The case scenarios used in this pilot were identified from the Lippincott VR-Sim library and adapted slightly to balance the difficulty level, that is, simple-to-complex concepts. Each scenario was also mapped to relevant entry-to-practice informatics competency indicators as they applied to the focus of the scenario [27].

A few days after completion of the 1-month pilot, the students were asked to complete the posttest survey and a satisfaction survey and to express interest in participating in semistructured focus group interviews to share their experiences. Two focus groups were scheduled to accommodate students’ schedules. The satisfaction survey included 15 items measured on a rating scale from 1 to 5 (1 being very dissatisfied and 5 being very satisfied). Some of these survey items were related to ease of use and user experience that, with permission, were slightly adapted from the DocuCare vendor product satisfaction survey. The remaining items related to perceptions of the impact on learning were added by the researchers. This survey was integrated as a complementary measure in case students were not able to participate in interviews because of their workload.

For nurse educators, a poster invitation was sent to recruit interested participants. Those who expressed interest in participating were offered a training webinar along with access to DocuCare and the same scenarios that students used for them to try out the product over a 2-week period and share their perspectives. Educators were also asked to express interest in participating in a focus group interview.

Each focus group interview lasted between 60 and 90 minutes. The interviewers (second and third authors) facilitated all discussions in English using an interview guide. Field notes were taken and used, where required, to assist with reflecting on the data during analysis. The discussions were audio-recorded and transcribed verbatim.

### Data Analysis

Data from completed surveys were included in the analysis, and incomplete surveys were excluded. Using IBM SPSS Statistics version 25.0, descriptive statistics, including frequencies, means, and SDs, were used to summarize and describe the data. A two-tailed paired *t* test using a difference score (posttest and pretest) was applied to test the null hypothesis: there was no statistically significant difference in students’ self-reported informatics knowledge and attitudes toward EHR mean scores before and after using the DocuCare program for 1 month (H0: M1−M2=0). For the qualitative interview data, the third author (UDW) compared all transcriptions and audio recordings to ensure the trustworthiness of the data. All transcripts were imported into the NVivo 11 data management software (QSR International Pty Ltd) and the data were coded and analyzed inductively. All authors discussed and defined emergent codes and themes to ensure intercoder reliability. The final codes and themes were refined until a consensus about the interpretations or coding frameworks was reached. Data were stored, managed, accessed, and analyzed within a secure SharePoint drive.

### Ethical Considerations

This study was approved by the institutional review and ethics board. Participation was voluntary, and students were assured that their participation had no impact on their academic performance. Each participant received an information sheet detailing the study procedures, benefits, and risks. Surveys were completed by implied covert action (ie, completion and submission of surveys). Written informed consent was obtained from all participants before the focus group sessions. Each interview began with an explanation of the purpose and procedures of the interview and assurance that the discussion would remain confidential.

## Results

### Characteristics of Participants and General Information on Informatics Learning

Although 23 students participated in this pilot study, the results are reported based on an analysis of 13 completed pretest-posttest surveys. This decision was made because of missing data between the pre- and posttest surveys. Most of these respondents represented 2 large undergraduate nursing programs: the Collaborative Program (n=7 students; 5 were third-year and 2 were second-year students) and the After-Degree Program (n=5; all were in their second year of study). Only one third-year student was from the honors program. There was no representation from first-year After-Degree students or those in the bilingual program.

Students (n=13) described their overall understanding of the concept of nursing informatics and its relevance to their future practice as moderate (4/13, 31%), fair (6/13, 46%), or inadequate (3/13, 23%). The majority indicated receiving specific learning about informatics competencies expected of registered nurses in Canada: information and knowledge management (12/13, 92%) and professional and regulatory accountability (13/13, 100%), but fewer reported on the competency pertinent to the use of digital health technology in clinical practice (10/13, 77%). With regard to learning about computerized documentation in EHRs during undergraduate education, 54% (7/13) of respondents indicated yes, whereas 46% (6/13) of them indicated no. Regarding permissions for students in the clinical setting, 54% (7/13) indicated they were permitted to view patient information with instructor or preceptor supervision. For permissions related to electronic documentation, almost two-third of the students (9/13, 69%) indicated that most clinical placement sites they went to did not use electronic documentation, whereas 23% (3/13) indicated yes and 8% (1/13) indicated sometimes.

### Self-reported Knowledge in Informatics and Attitudes Toward EHRs

Table 1 provides an overview of the mean difference scores for the pre- and posttest surveys. The two-tailed paired samples *t* test to evaluate the impact of DocuCare on students’ self-reported informatics knowledge and attitudes toward the EHR revealed a statistically significant difference in the mean score of knowledge before (mean 2.95, SD 0.58) and after using DocuCare (mean 3.83, SD 0.39; t_12_=5.80, two-tailed; *P*<.001). The mean score was −0.88 (SD 0.54) with a 95% CI ranging from −1.21 to −0.55. The eta-squared statistic (0.74) indicated a large effect size. Therefore, the null hypothesis was rejected. However, there was no statistically significant difference in the mean scores of attitudes toward the EHR before (mean 3.75, SD 0.40) and after using DocuCare (mean 3.70, SD 0.34; t_12_=0.39, two-tailed; *P*=.70). The mean score was 0.05 (SD 0.43), with a 95% CI ranging from −0.21 to 0.30. Therefore, we failed to reject the null hypothesis, that is, there was no statistically significant difference in students’ self-reported informatics attitudes toward the EHR mean scores before and after using the DocuCare program.

**Table 1:** Self-reported informatics knowledge and attitudes toward electronic health records before and after using DocuCare (N=13).

Items			Pretest, mean (SD)		Posttest, mean (SD)		Difference score		*P* value
**Informatics knowledge: I have...**									
	A good knowledge to critically evaluate data and information from a variety of credible sources (including experts, clinical applications, databases, practice guidelines, relevant websites, etc) to inform the delivery of nursing care.	4 (0.577)		4.15 (0.555)		−0.154		.16	
	A good knowledge of the various components of the EHR^a^ such as results reporting, clinical documentation, electronic medication administration, etc).	2.62 (1.044)		3.77 (0.439)		−1.154		.003	
	A good knowledge related to documenting important nursing and patient data using standardized nursing languages, such as the International Classification for Nursing Practice, ie, nursing diagnosis and interventions to support clinical decision-making and nursing practice improvement.	2.85 (0.987)		3.54 (0.877)		−0.692		.08	
	A good knowledge related to gathering and retrieving relevant health care and patient data from the EHR.	2.77 (1.092)		3.92 (0.494)		−1.154		.001	
	A good knowledge related to documenting health care and patient data into the EHR.	2.54 (1.198)		3.77 (0.439)		−1.231		.004	
**Attitudes toward EHRs**									
	Use of EHRs are more of a help than a hindrance to patient care	3.85 (1.214)		3.62 (1.121)		0.231		.68	
	Use of computerized charting has helped to improve documentation of the clinical record	3.92 (0.760)		4 (0.913)		−0.077		.78	
	EHRs pose less threat to the patient’s privacy than do paper records.	3.23 (0.599)		3.08 (0.954)		0.154		.44	
	Computerized charting has decreased the workload of nurses and other personnel.	3.62 (0.768)		3.54 (0.660)		0.077		.58	
	In time, the use of EHRs will lead to improved patient care	4.15 (0.376)		4.31 (0.480)		−0.154		.16	

^a^EHR: electronic health record.

### Accuracy of Students’ Electronic Documentation

For most students, their scores improved as they progressed from case scenarios 1 to 4, with more practice and ongoing feedback. Of the 13 students, 9 completed the 4 case scenarios. The lowest total score obtained by these students was 11 of 16, whereas the highest score was 15.

### Students’ Satisfaction With the DocuCare Learning Experience

Of the 13 student participants, 12 (92%) completed the satisfaction survey at the end of the pilot. Only 42% (5/12) of these students attended the in-person orientation. Of these 5 students, 3 (60%) were satisfied with this session. Regarding the frequency of accessing DocuCare during the pilot, 58% (7/12) of students indicated accessing the program once a week, 25% (3/12) accessed it multiple times each week, and 17% (2/12) accessed it once a day or multiple times each day. Most students (11/12, 92%) were satisfied to very satisfied with the ability to access DocuCare at any time and from anywhere, the ease of use when submitting completed learning activities for assessment, and the ability to learn at their own pace. In addition, students were satisfied to very satisfied with the DocuCare layout (8/12, 67%) and reported that they encountered no technical glitches when using DocuCare (7/12, 58%).

With regard to learning, two-thirds of the students (9/12, 75%) were satisfied to very satisfied that DocuCare provided a realistic learning experience about computerized charting in EHRs because, in principle, it was similar to the electronic health or patient records used in the clinical setting. It also helped increase their confidence in gathering relevant patient information to inform clinical judgment and nursing care planning for the assigned case scenarios used in this pilot study and care planning in general. The majority of the students (11/12, 92%) were also satisfied to very satisfied that DocuCare increased their critical thinking and clinical judgment abilities and their overall confidence in applying computerized charting in a real clinical setting (10/12, 83%). Overall, 92% (11/12) of students were satisfied to very satisfied that using DocuCare enabled them to progressively enhance their informatics competencies and recommended using it for students’ learning about computerized charting in undergraduate nursing programs.

In their responses to an open-ended question at the end of the satisfaction survey that asked what was helpful and what could be improved, the students provided some insights. One student indicated:

It could’ve been better if I attended the orientation session prior to the study as the program was hard to use in the beginning. However, the program allowed me to find the areas which I need to improve on such as critical thinking skills and judgement. I think it will be helpful/beneficial to students if the DocuCare program becomes part of the school curriculum.

Another student asserted that DocuCare was user friendly:

it was nice and simple to follow through with the steps, and the feedback was really helpful as well.

Another student added:

definitely getting that feedback..., that did help me increase my confidence in what I could do on this system, so I thought that was helpful in that sense.

### Focus Group Interviews With Students and Faculty

A total of 7 students participated in focus groups, one interview had 4 students whereas the other had 3 students. A total of 7 educators attended the DocuCare webinar and tested the product; however, only 3 educators expressed an interest and participated in the focus group interview. Participants described their experiences using DocuCare as a learning tool to improve computerized documentation and overall informatics competency. Overall, 5 themes emerged, which were as follows: (1) current challenges related to documentation, (2) motivating factors for embracing computerized charting as an educational tool, (3) educators’ and students’ experiences with DocuCare, (4) educators’ and students’ suggestions for improving DocuCare, and (5) recommendations for integrating DocuCare in undergraduate nursing curricula.

#### Theme 1: Current Challenges Related to Documentation

Reflecting on teaching and learning experiences in clinical practicum sites and acknowledging that they may become more complicated with the ongoing Connect Care implementation, the participating educators and students agreed that a number of challenges related to documentation currently exist in clinical and laboratory learning. This first theme, *current challenges related to documentation*, comprised 6 categories, 4 of which were exclusively from educators’ perspectives: students’ level of experience, stressful clinical settings, student-instructor ratios, and limited laboratory training hours. The following excerpt illustrates educators’ concerns about students’ level of experience and student-instructor ratios:

For some of them, they’ve never been in the hospital, so it’s a really, you know, unfamiliar environment, and it is really high stakes. They’re real people involved. You know, and so their stress levels are already really high...I think...if you have eight students and one instructor to do some of that stuff on the fly, like with labs we have to do sometimes, is incredibly stressful for the instructor and really stressful for the students.

Educator 3

A number of educators also underlined the limitations in laboratory training hours and in teaching about documentation and informatics as obstacles they currently encounter in clinical practice:

we have two-hour labs now—we don’t always have a lot of time for them to sit down and do a narrative charting of what they just did

Educator 2

In addition, educators felt that the stressful clinical setting compounded the challenges faced by students when learning about documentation:

you get in front of the computer...it is the time factor...they get their two minutes on there, because everybody else is lined up for them, right...and when they get into a hurry, mistakes get made...they’re charting on the wrong person and everything, getting everybody stressed out...and I just find even with the long paper charting, the entry “I received into care, blah blah blah, found sitting, breathing normally,”...they’ve got this whole thing memorized, so they’re not even critically thinking about that.

Educator 1

Similarly, students shared their views on the challenges encountered with learning about documentation, both in theory and in a clinical setting. The limited teaching on documentation and informatics was perceived as a substantial challenge:

I do remember having a couple classes maybe where they taught us about charting in first year—which, I mean, it felt really awkward to do it, but I think—they did kind of tell us the basics. But yeah, I feel it’s just kind of an awkward process of learning how to do it properly

Student 1

Students also mentioned their stressful experiences of learning through trial and error in clinical sites:

...going through this program one of the biggest struggles that I’ve had was documentation, period. Whether it was on paper or in—that’s one thing that I felt could ’ve been addressed a little bit more...my very first clinical, I had no idea. Like, not a clue. So, I’m just copying from what my nurse that was buddying me, what they did—I mean, it may have been right, it might’ve been wrong, but that’s the tool that I had at that time. As time has gone on, I’ve kind of figured out different ways to do things.

Student 2

#### Theme 2: Motivating Factors for Embracing Computerized Charting as an Educational Tool

Participants’ reflection on informatics and DocuCare as a simulated record assisted in creating the second theme: *motivating factors for embracing computerized charting as an educational tool*. This theme included 3 categories: ideal timing, fostering exposure, and learning opportunity.

Participants acknowledged the need to prioritize the introduction of EHRs during their nursing education. They felt that their time in school was the ideal time to learn about EHRs because of their availability and openness and the school’s propitious learning environment. According to one student:

I think that informatics in general is like, a whole concept, a language, a culture, a mindset—and you have to learn how to use that...I think it’s very important. If we’re using these kinds of systems, I think this is the perfect time to teach that. It’s a time when we’re not as worried about maybe—other job or political ramifications—we’re here at school to learn and to be educated, and to prepare for that setting. So, I think this is a captive audience who wants to learn, and that means it’s a perfect time. And if we are here for two to four years, that’s enough time that we can go and ask more questions about it, and kind of say what we want to see better or worse, whereas in—afterwards, time sometimes goes a little bit faster. So, I think the fact that we’re here and we’re learning about the job—this is in part—a huge aspect of the job, and it’s a perfect time to do it.

Student 2

Educators followed up by emphasizing that fostering *hands-on* experience in simulated electronic records is vitally important for the development of documentation competencies and for building confidence in using clinical information systems in clinical settings:

This will just open their eyes to a bit of what they can expect...documentation...it’s so different than, you know, they write essays. But charting is not like that...it’s like a new language almost for them, right...it’s such a looming thing you know at the beginning for them that I think the more that they can kind of get exposed to it and see it and get familiar with in ways that sort of—you know, the more we can kind of tie as many pieces together as we can early on...you know, the better.

Educator 3

Similarly, students shared important insights into how fostering exposure with hands-on practice opportunities with simulated records may help provide a standardized approach to refine documentation skills, alleviate anxiety, and enhance overall readiness for clinical practice:

So, I felt very disorganized going to clinical, even though I was trying to follow that paper, and I know it’s somewhat charting by exception, but—I think with DocuCare it will definitely help me organize my thoughts better—kind of understand the assessments and questions they would ask, and to use that to develop other like, nursing diagnoses. And how to interpret other kind of orders that are put in place better...I would say it [DocuCare] definitely gave me a bit of perspective of what using digital health resources means, like in terms of navigation, in terms of like—all those little details and like, the potential value of it.

Student 3

Several of the participants highlighted that there were valuable learning opportunities, especially those regarding the refinement of documentation skills and enhancing their critical thinking. One student stated the following:

The learning was multifaceted for me—it did bring up that systems analysis part, like—what would be the challenges of implementing this? What would be the challenges of me using this as a clinician on the floor? What are the challenges, how do they affect the patient? I don’t understand why wouldn’t try to teach the exact programs that we’re trying to implement in the hospital so that there’s a lot more confidence when you’re graduating nurses. I’d like to know how to do it, like—have a computer that you’ll have on a unit in the lab, so that you’ll have a chance to see what it feels like to document.

Student 1

Furthermore, participants agreed that the learning opportunities offered while using DocuCare would be transferable to different EHR systems. This transferability would occur in a learning juxtaposition of facts and theories being formed during school with DocuCare and the new systems and processes they encounter when they become clinicians, which may result in augmenting or restructuring the former. One educator certified the following:

I work on so many different units, and every chart is set up a little bit differently, and things are found in slightly different places...I guess going from DocuCare to Connect Care, there might be a little bit of an adjustment if they’re slightly different, but really once they’re on Connect Care and familiar with where everything is, it’ll be the same no matter where they go, which’ll be great. [The systems] are both different, but I think—it’s similar enough...we teach our students critical thinking, that sort of thing—like I actually was really impressed with how easy I found this. And I think because—even though they’re not exactly the same, they’re similar enough.

Educator 3

Students corroborated educators’ views regarding learning opportunities and the transferability of knowledge, where simulated learning with DocuCare may help address gaps and inconsistencies relevant to the limited opportunities for learning about clinical informatics systems. This was evident for students who had opportunities to receive Connect Care training before the pilot. They were able to compare similarities and differences and think about how these systems can be used in a complementary way to address gaps in students’ learning. As a student explained:

I did start off with Connect Care before I started the DocuCare so, in some sense, I could see where I’m navigating, but likewise I’ve never done orders and stuff on Connect Care—I just know the basic assessments and stuff. And like, I do see differences—Connect Care is more advanced with its formatting, navigating through tabs and the information they portray, but I think DocuCare is a great kind of preliminary health record for students to learn if they can’t have access to Connect Care yet—because Connect Care is very limited in locations, and as they slowly roll it out, whereas DocuCare is a—kind of a good simulation to get comfortable with, you know, the electronic health record.

Student 2

#### Theme 3: Educators’ and Students’ Experiences With DocuCare

During the focus group interviews, participants described their experiences with DocuCare. On the basis of the accounts that emerged, we attempted to understand the experiences with DocuCare for both students and educators. This third theme is divided into 4 categories: ease of use, provision of feedback to students, well-integrated layout, and tools to provide safer care.

Although educators did not complete a satisfaction survey, they shared important insights through the focus group interviews. Educators described DocuCare as easy to use and having many features that can facilitate engagement with learners:

I think it was pretty easy to go in and find the tabs, and then you’d find something else the next time you went in that you missed last time, so—it was just a matter of spending that time doing it, right?

Educator 1

Educators also mentioned the DocuCare feature for providing feedback to students on their submissions was seamless and efficient, thus promoting learning and positive interactions with students. As an educator indicated:

I thought it was kind of easy because you could just go in there and say, Okay, incomplete assessment, now please resubmit...It was non-offensive, supportive, which is sort of what we do, and then there’s a box to add your comments

Educator 1

Educators also found that the layout of tabs within DocuCare was well integrated and presented a comprehensive view of the clinical information that students need to document, and it included a review of the care provided. According to one educator:

Well, definitely the tabs. I did like that you had your flow sheets; you had your assessment...you look at it...and it’s there. You don’t have to go fishing around, it’s a click. The layout, the dropdown menus—I mean everything about it is very familiar, I didn’t have a hard time navigating it.

Educator 2

Students also stated that DocuCare reinforced the provision of safer care. According to them, safer care was linked to improvements in communication, efficiency, evidence-based practice, and humanization of care. One student noted:

The doctor’s writing is really confusing, and I think if you were to consolidate all of that onto electronic charting, it’d be a lot easier to follow, like, the story of how they’ve been doing up until like the point that you’re now taking over...In some situations, it [DocuCare] does condense the information down and get you more focused on what’s important, what’s necessary—and as well for multiple people, it kind of makes everybody on the same field. So, somebody might write something or explain something in a completely different way, but this way—it’s all uniform almost. So, it’s harder for me to get like, a full picture of the patient, whereas with the DocuCare it was actually—once I figured out where to go—it was pretty easy to see, Okay, this is how they were on their last shift, because it’s just typing and everything comes out really clear, because it’s electronic. So that I actually found it really easy in terms of finding like, past information.

Student 2

#### Theme 4: Educators’ and Students’ Suggestions for Improving DocuCare

Many responses were assigned to the theme *suggestions for improving DocuCare*. This theme, which is concerned with suggestions for improvement, is divided into 3 categories: warning signs, search functions, and indexing patient information. Educators suggested that it might be beneficial to have a warning sign within DocuCare, allowing users to know when the data chart is incomplete. Educators clarified that they (students):

always forget something when they do an assessment...it would be kind of nice if it glowed or something—it wouldn’t let you carry on unless you complete it

Educator 1

Conversely, students felt that having a search function in DocuCare would make it more user friendly and easier to navigate. One student explained:

In this world we all know that there’s so much information, it’s changing all the time, and it could be found anywhere. If I would’ve had a search bar where I could have typed in “urticaria,” that would’ve brought up which sections are those in...it was very inefficient for me to spend an hour trying to look for something. So, if there’s a tool that allows me to search for it quick, then—I think that would be super helpful.

Student 1

Other students suggested adding a sidebar indexing patients’ most important and recent information would enhance access to the most relevant information needed when providing care within DocuCare. This indexing function is actually similar to what is typically seen in clinical sites. According to one student:

With Connect Care they have the patient’s information consistently on the side. You can easily access the main information that you’re reporting, like, what’s their weight and diet, what’s coming up for them...so you can view everything, and even vital signs. So, where DocuCare is very fragmented in different tabs, Connect Care has it in like an actual flow sheet, so where you know, you just read, scroll, and you can add it in at the same time.

Student 3

#### Theme 5: Recommendations for Integrating DocuCare in Undergraduate Curricula

This theme comprised recommendations for integrating DocuCare in the undergraduate nursing curricula. Specifically, it included 4 categories: better orientation and support, more exposure and frequency, scheduling DocuCare in the curriculum and integrating it within courses, and flexibility of delivery with a focus on learning, not grading.

Both educators and students agreed that the introduction of DocuCare in the undergraduate curricula should start with an orientation for both educators and students on the system usage coupled with ongoing technical and human support. According to one student:

It’ll be even good to just have like, one video example with like, the computer videos of a teacher or instructor just going through one basic assessment, or one case scenario, so students can visualize and see—This is how it [kind of] should be done.

Student 3

Educators, conversely, found that the orientation session provided to them was sufficient:

I found the webinar actually really helpful. After I watched that I was like, “Ah, piece of cake!” You know—It was not intimidating at all!

Educator 2

However, educators felt that:

a designated tech support person would be good

Educator 2

and:

having access, like—on [our] time, because setting things up...so, if there’s a way that we could have sort of continuous access to it, that would be—I think better

Educator 3

Educators and students also agreed that incorporating DocuCare in the education of nursing students should be systematic, with more frequent exposure. They suggested that exposing students to it in the simulation laboratory and, where possible, in clinical settings would allow adequate opportunities for students to develop proficiency and integration with their overall repertoire of clinical skills. One student pointed out:

Yeah, and I mean even still at the end of all the cases that I did—I still think I wasn’t, I still would’ve wanted a little bit more practice. If that was my method of documentation on the floor, I would definitely want more than what I got in the cases that I did...I think that it would be beneficial to do it throughout the program, not just a one-time thing, and kind of starting it off slow or starting it off smaller, and then growing the pieces bigger so that you are constantly exposed to it and constantly practicing through it.

Student 2

Students and educators differed in their views about the timing of introducing DocuCare in the curriculum and integration with other courses. For students, starting early in their program would be most useful; however, they believed that having some foundational knowledge in nursing first would be more appropriate. According to one student:

I think—maybe at the end of first year, but more in second year, because I think for me, like figuring out how to do the assessments first—instead of having like—learning to do the assessments, and then learning to use DocuCare—I think I want be more comfortable with actually what I’m doing before I try putting in...[Student 1]

Conversely, educators felt:

it would be really valuable, that first year in health assessment, if we can give them time

Educator 2

Finally, educators and students provided important insights on how DocuCare learning could be incorporated despite challenges with content-heavy curricula and busy students’ schedules. Flexible delivery, with a focus on learning not grading, was highlighted. Students highly supported the use of self-directed learning via online modules that can be incorporated into the laboratory or e-class platform without imposing a grading system. According to a student:

I could even see it as an e-class module...and with the no marks thing. I think it’d be good if simulation lab had an opportunity to like—you go in, here’s your patient, here’s your thing. But I think it’d be great if there was an option—almost like a little certificate kind of thing. You could come at your own time, here are the times and dates, we have the operator here, here’s a case scenario, we need you to come in maybe with yourself or with a partner, or even as a clinical group.

Student 3

Conversely, educators felt the need to engage students in this learning and to monitor their progress down the road:

You have to make them accountable in some way...you just put pass or fail.

Educator 3

## Discussion

### Principal Findings

The purpose of this pilot study was to determine feasibility issues and evaluate the suitability of DocuCare from the perspectives of students and educators, as a tool for supporting students’ acquisition of informatics competency and to enhance their readiness for future practice in digital health environments. The use of DocuCare in this study contributed to improving students’ knowledge about informatics and accuracy of electronic documentation, congruent with findings from previous research [16,19,24,40-42,47]. There was no change in attitudes toward EHRs. On the basis of the findings from focus group interviews with students, this could be explained by the fact that students already had positive attitudes toward this technology, as demonstrated in their responses. In our study, educators agreed that *hands-on* learning opportunities through simulated electronic records were vitally important for the application of knowledge, development of critical thinking and documentation competencies, and building confidence in using clinical information systems in practice settings. Similarly, students were overwhelmingly satisfied to very satisfied with DocuCare and highly recommended using it in undergraduate nursing programs. They indicated that opportunities for electronic documentation will not only strengthen their communication skills but also enhance their critical thinking skills and their understanding of informatics concepts. It could ease their transition to using advanced technology in the work setting, which is congruent with findings from previous research [11,47].

The results of this study and other research confirm that students recognize gaps in their informatics preparedness to meet workplace requirements regarding the use of electronic tools to support nurses’ work, particularly for data management and electronic charting [5,6,11,16,42]. Uniquely, in our study, students and educators provided suggestions for improving the DocuCare platform. They also shared strategies and recommendations on how DocuCare could be incorporated in strategic ways to augment students’ learning about digital health and informatics without creating additional workload or overwhelming the already content-heavy curricula. Students were also quite passionate and willing to take on a self-directed role in embracing this technology to support their education. They recognize that the technological changes taking place in the clinical environment warrant action, but their education was not keeping pace. Although these findings reflect more awareness among student nurses regarding the importance of digital health and informatics in nursing practice, they also assert the need for more work on the part of nursing schools and nurse educators in creating learning opportunities within the curriculum to enhance students’ preparedness for their future nursing roles. Unfortunately, within Canadian prelicensure education, significant gaps still need to be addressed.

In a recent survey of Canadian schools of nursing, fewer than 20% of 360 nurse educators surveyed reported using EHR simulation in conjunction with teaching clinical skills in a simulation laboratory [5]. Congruent with these findings, in another survey conducted by Canada Health Infoway, only 35% of the nurse educators surveyed reported using a training version of an electronic record or clinical information system to support the teaching of nursing skills [2]. In addition, despite the increased utilization of digital health tools in practice, only 6 of 10 nurses surveyed about the use of virtual care reported having adequate knowledge and skills to use these systems [2].

Nagle et al [5] identified that teaching students about the use of EHRs is challenging in practice settings because there are few opportunities for students to access or use fully functional EHR systems in health care facilities. These findings are congruent with our findings and those in the general literature [11,34]. Nagle et al [5] also highlighted the need for an affordable EHR *sandbox* that could be used in simulation laboratories to teach students how to integrate these tools as they learn other clinical skills. They also identified the need for academic administration support for nurse educators as they implemented these tools [5,54]. Simulated EHRs are valuable for students learning about informatics and computerized charting; therefore, removing barriers to integrating them within Canadian nursing education should be a priority [5,20,34,42,54].

Educating future nurses about digital health and informatics is no longer an option but rather a core requirement in modern-day nursing practice [5,34,40,42,54-59]. Although the abrupt transition to remote delivery of education during the COVID-19 pandemic highlighted the current gaps in technology infrastructure and created challenges for higher education institutions worldwide, it also revealed opportunities for embracing technology and virtual simulation. This could be an important opportunity for nursing programs to capitalize on as we navigate more virtual practice across all domains of nursing [60-64].

### Limitations

As this was a pilot study and voluntary participation was appreciated, the small sample size and the use of a one-group quasi-experimental design affected the power of the study. Numerous contextual factors may also have influenced students’ willingness to participate in the study. The participants’ recruitment was interrupted because of the COVID-19 pandemic and the public health guidelines restrictions. Students’ academic workload, disruption of clinical placements, stress and feelings of uncertainty related to the COVID-19 pandemic, and the uncertainty regarding the completion of their program and entering the workforce in an evolving global pandemic may have also influenced students’ desire to participate in this research. Finally, students who agreed to participate might have been more interested in the topic of the study compared with those who did not participate, which may have resulted in a response bias.

### Conclusions

The integration of simulated EHRs within the nursing curriculum has the potential to improve students’ knowledge and understanding of informatics and build confidence in using EHRs, including computerized charting. In this pilot study, the opportunity to use DocuCare firsthand enabled our students and educators to provide important insights and recommendations to the curriculum committee on the suitability and value of this educational tool for improving teaching and learning about informatics, computerized documentation, and the use of EHRs. This preliminary evaluation will also inform the planning of a future larger, controlled evaluation study, inviting students and educators from other sites within our collaborative nursing programs in Alberta. Given the paucity of Canadian research on simulated EHRs, the findings of this study may also be useful to other schools of nursing.

## Abbreviations

EHR: electronic health record
EMR: electronic medical record
